# Care of elderly patients: a prospective audit of the prevalence of hypotension and the use of BIS intraoperatively in 25 hospitals in London

**DOI:** 10.1186/s13741-016-0036-1

**Published:** 2016-05-27

**Authors:** Alex Wickham, David Highton, Daniel Martin, The Pan London Perioperative Audit and Research Network (PLAN)

**Affiliations:** Department of Anaesthetics, Imperial College Healthcare NHS Trust, London, UK; Neurocritical Care, the National Hospital for Neurology and Neurosurgery, University College London Hospitals, Queen Square, London, UK; Division of Surgery and Interventional Science, Royal Free Hospital, University College London, Pond Street, London, NW3 2QG UK; Royal Free Perioperative Research, Department of Anaesthesia, Royal Free Hospital, Pond Street, London, NW3 2QG UK; The London Academy of Anaesthesia, London, UK

**Keywords:** Elderly, Intraoperative hypotension, Depth of anaesthesia

## Abstract

**Background:**

Anaesthesia is frequently complicated by intraoperative hypotension (IOH) in the elderly, and this is associated with adverse outcome. The definition of IOH is controversial, and although management guidelines for IOH in the elderly exist, the frequency of IOH and typical clinically applied treatment thresholds are largely unknown in the UK.

**Methods:**

We audited frequency of intraoperative blood pressure against national guidelines in elderly patients undergoing surgery. Depth of anaesthesia (DOA) monitoring was also audited due to the association between low DOA values and IOH with increased mortality (as part of “double” and “triple low” phenomena) and because it is a suggested management strategy to reduce IOH.

**Results:**

Twenty-five hospitals submitted data on 481 patients. Hypotension varied depending on the definition, but affected 400 patients (83.3 %) using the AAGBI standard. Furthermore, 2.9, 13.5, and 24.6 % had mean arterial blood pressures <50, <60, and <70 mmHg for 20 min, respectively, and 136 (28.4 %) had systolic blood pressure decrease by 20 % for 20 min. DOA monitors were used for 45 (9.4 %) patients.

**Conclusions:**

IOH is common and use of DOA monitors is less than implied by guidelines. Improved management of IOH may be a simple intervention with real potential to reduce morbidity in this vulnerable group.

## Background

Older patients are vulnerable to perioperative complications due to a combination of physiological frailty and comorbid disease and now constitute an increasing proportion of the surgical population (Griffiths et al. 2014). Changes in intraoperative haemodynamics and oxygen delivery have been a focus of interest in the mechanism of perioperative organ dysfunction (Lobo and de Oliveira 2013) and therefore may be of increased relevance in older patients. Blood pressure and depth of anaesthesia influence organ (particularly cerebral) perfusion and oxygenation and are routinely manipulated intraoperatively. Recently, this has been a focus of interest in the Anaesthesia Sprint Audit of Practice (ASAP) (ASAP collaboration team 2014) highlighting a high prevalence of intraoperative hypotension (IOH) in patients who sustained fractured necks of femur.

Hypotension occurs frequently during anaesthesia and is associated with adverse outcomes in the elderly. These include stroke, compromised postoperative neurological performance, acute kidney injury, myocardial infarction, and increased 30-day and 1-year mortality (Bijker et al 2009; Bijker et al 2012; Yocum et al. 2009; Sun et al. 2015; Walsh et al. 2013; Monk et al 2005; Mascha et al 2015). Thus, the Association of Anaesthetists of Great Britain and Ireland (AAGBI) guidelines recommend IOH should be avoided in older patients (Griffiths et al. 2014), although the specific thresholds that constitute hypotension remain a topic of considerable debate (Bijker et al. 2007; Warner and Monk 2007; Brady and Hogue 2013). Both the prevalence of hypotension and associated complications vary widely in the literature, in part due to the range of threshold values used to define hypotension. The AAGBI recommend avoiding >20 % drop in systolic blood pressure (Griffiths et al. 2014), whilst drops of >30 % mean arterial pressure (MAP) and MAP <55 mmHg have been associated with stroke, myocardial ischaemia, and kidney injury (Bijker et al. 2009; Walsh et al. 2013). The treatment threshold applied in clinical anaesthetic practice in the UK is largely unreported, and this is key to addressing improvement.

Excessive anaesthetic depth is implicated in the mechanism of haemodynamic compromise, has been associated with myocardial infarction and stroke, postoperative delirium and, when combined with hypotension and low inspired anaesthetic concentration, increased mortality (Leslie et al. 2010; Radtke et al 2013; Sessler et al. 2012; Willingham et al. 2014; Willingham et al. 2015), a phenomenon referred to as the ‘triple-low’. Patients exhibiting the triple low for 60 min or longer are a particularly high risk group, with a quadrupled risk of 30-day mortality. The National Institute for Health and Care Excellence (NICE) recommends using electroencephalogram (EEG) based DOA monitors for patients at ‘higher risk of adverse outcomes’, specifically citing ‘older patients’ (National Institute for Health and Clinical Excellence 2012), and this might be a means to reduce the risk of haemodynamic compromise.

Maintaining intraoperative blood pressure and depth of anaesthesia within broadly normal limits are thus generally accepted standards, supported by recent evidence and national guidelines, and might influence outcomes for elderly patients. It is known that established standards are not met in the hip fracture population (ASAP collaboration team 2014), yet the frequency of such deviations is unknown in other older surgical populations in the UK. The aim of this audit was to characterise the incidence of IOH and the use of DOA monitoring in older surgical patients against current standards. A secondary goal was to assess the feasibility of the newly formed trainee-led Pan London Perioperative Audit and Research Network (www.uk-plan.net). The latter methodology is part of a national programme of network-based, trainee-led collaborations that aim to deliver audit, research and quality improvement projects.

## Methods

A prospective snapshot audit was conducted across 25 hospitals in London within the PLAN network. The project was confirmed to be a clinical audit by Imperial College Healthcare NHS Trust Clinical Governance department; they confirmed that research ethics committee approval and individual patient consent were not required because it is an audit and not research. Appropriate approval for the audit was obtained from clinical governance departments locally at each participating hospital.

Patients aged 65 years or older having a surgical procedure under general anaesthesia were included. Patients receiving sedation or regional anaesthesia as the sole method of anaesthesia, intracranial neurosurgery, cardiopulmonary bypass (non-pulsatile hypotension implicit) or jet ventilation were excluded. Audit standards and definitions were identified from consensus statements, research and national guidance. The AAGBI standard, ‘a fall in systolic blood pressure of more than 20 % from pre-induction baseline… is a suitable limit’ (Griffiths et al. 2014), was used for IOH. The standard for DOA monitoring, ‘the use of EEG based depth of anaesthesia monitors is recommended… in patients at risk of adverse outcomes’, was taken from NICE guidelines (National Institute for Health and Clinical Excellence 2012).

Data was collected from the anaesthetic record by anaesthetic trainees who were independent of the clinical care of the patients over two locally determined weekdays in July and August 2014. To ensure no cases were missed, trainees collected data on ‘non-clinical’ days or used study leave and worked closely with nursing staff in each recovery area of their hospital. All patients passing through recovery were assessed for inclusion. Data were collected using a paper case report form.

Blood pressure values were recorded from the anaesthetic chart (either handwritten paper forms, or an electronic print out or log), which are typically documented every 5 min. The values were taken at 5-min intervals for both non-invasive and invasive monitoring. Preoperative blood pressure was determined using a reading from pre-assessment clinic, the theatre care plan or ward observation chart (last recorded value). Pre-induction blood pressure documented was the blood pressure taken immediately prior to the induction of anaesthesia. Where multiple hypotensive episodes occurred, total cumulative hypotensive time was calculated. If different degrees of hypotension occurred at separate time points, the lowest value was taken to calculate the degree of drop from the pre-induction pressure. The frequency of recording DOA values varies, but is typically every 15 min. Types of DOA monitoring and cumulative duration of scores less than 40 were documented.

Statistics were calculated using R version 3.2.0 (© 2015 The R Foundation for Statistical Computing). Data were examined for normality using the Shapiro-Wilk test. Paired data was compared using the Wilcoxon matched pairs test. Categorical data analyses were made using chi square test with Fisher’s correction. All tests were two tailed, and significance was taken as *p* < 0.05. Continuous data is presented as median (IQR [range]), categorical data as number (proportion).

## Results

Twenty-five hospitals submitted data for 481 patients. The median number of patients per hospital was 19 (IQR 16–23, range 7–60). Results were received from 11 (44.0 %) district general hospitals, 11 (44.0 %) teaching hospitals and 3 (12.0 %) single specialty hospitals. One case was not completed beyond detail on gender and was therefore excluded from analysis. Paper-based anaesthetic records were used in 411 (85.6 %) cases whilst printed computerised records were available in 6 (24.0 %) hospitals and therefore used for 69 (14.4 %) cases. Incomplete recording of blood pressure was present in 12 cases on paper notes (2.5 %) compared with 0 cases with an electronic record. The patient and surgery characteristics, frequency of systolic hypotension and prolonged IOH are shown in Table 1.

**Table 1:** Patient characteristics, frequency of intraoperative systolic hypotension (≥20 % from pre induction value) and prolonged hypotension. Values are number (percentage)

Variable		Patient characteristics	Frequency of systolic hypotension	Frequency of systolic hypotension lasting >20 min
Gender	Female	234 (48.9)	201 (85.9)	67 (27.4)
	Male	245 (51.2)	197 (80.4)	74 (31.6)
Age	65–74	286 (59.5)	244 (85.3)	100 (35.0)
	75–84	152 (31.6)	119 (78.3)	35 (23.0)
	>85	43 (8.9)	35 (81.4)	6 (14.0)
Surgical speciality	General, breast and endocrine	188 (9.1)	158 (84.0)	48 (25.5)
	Orthopaedics, trauma, plastic and spinal	145 (30.2)	115 (79.3)	37 (25.5)
	Other	36 (7.5)	29 (80.6)	11 (30.6)
	Gynaecology	35 (7.3)	29 (82.9)	15 (42.9)
	Vascular	25 (5.2)	23 (92.0)	12 (48.0)
	ENT	22 (4.6)	20 (90.9)	8 (36.6)
	Thoracic	18 (3.7)	13 (72.2)	7 (38.9)
	Ophthalmology	12 (2.5)	12 (100.0)	3 (25.0)
NCEPOD classification	Immediate	5 (1.0)	4 (80.0)	1 (20.0)
	Urgent	64 (13.3)	57 (89.1)	15 (23.4)
	Expedited	32 (6.7)	24 (75.0)	13 (40.6)
	Elective	379 (79.0)	314 (82.9)	113 (29.8)

The method for measuring intraoperative blood pressure was recorded in 478 cases (99.4 %), and 89 (18.6 %) patients in this group had invasive monitoring. Preoperative blood pressure values were not documented in 47 cases (9.8 %). Stage 1 hypertension (BP >140/90 mmHg (National Institute for Health and Clinical Excellence 2011) was present in 38 (7.9 %) patients preoperatively. Although the systolic blood pressure was significantly higher pre-induction (*p* = 0.02), the effect size was small, median preoperative blood pressure 140/77 mmHg, and pre-induction 140/78 mmHg (Table 2).

**Table 2:** Preoperative, pre-induction and intraoperative blood pressure. Values are median (IQR [range])

	Preoperative (mmHg)	Pre-induction (mmHg)	Lowest intraoperative (mmHg)	Difference between preoperative and pre-induction value (Wilcoxon matched pairs)
Systolic	140 [125–151 (90–209)]	140 [125–155 (80–215)]	90 [80–100 (46–157)]	*p* = 0.02
Diastolic	77 [69–85 (23–114)]	78 [68–85 (38–178)]	50 [42–58 (25–94)]	*p* = 1.00

**Fig. 1:**
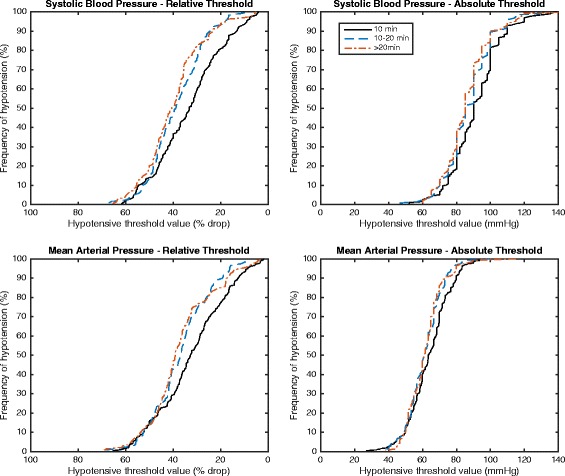
Frequency of hypotension as a function of the definition used

Figure 1 illustrates the frequency of hypotension as a function of the definition used, and it can be seen that this causes considerable variation in the reported IOH. Table 3 details the prevalence of IOH using different thresholds: 0–20.0, 20.1–40.0 and ≥40.1 % drop from pre-induction baseline systolic blood pressure. Hypotension of more than 20.0 % drop occurred in 400 patients (83.3 %), and in 188 (39.2 %), the drop was ≥40.1 %. In 141 (29.4 %), this lasted for longer than 20 min. Systolic hypotension (>20 % drop) occurred across all patient age groups (65–74, 75–84, ≥85 years old), in all surgical specialities and in both elective and unscheduled surgery (see Table 1). Lowest reported intraoperative systolic (87 versus 80 mmHg) and mean (61 versus 57 mmHg) blood pressure values were significantly lower in patients whose blood pressure was recorded electronically compared with those whose blood pressure was handwritten (*p* < 0.001 and *p* = 0.005, respectively); 129 (26.8 %) patients had mean arterial pressures less than 55 mmHg at some point during their surgery; this persisted for over 20 min in 45 (9.6 %) patients.

**Table 3:** Duration of intraoperative systolic hypotension. Data was available for 461 cases. Values are number (proportion). Drop defined from pre-induction systolic blood pressure

Drop in systolic blood pressure	<10 min	10–20 min	>20 min	Total
0–20.0 %	54 (11.37 %)	7 (1.5 %)	5 (1.0 %)	66 (13.8 %)
20.1–40.0 %	82 (17.1 %)	57 (11.9 %)	68 (14.2 %)	207 (43.1 %)
>40.1 %	60 (12.5 %)	60 (12.5 %)	68 (14.2 %)	188 (39.2 %)
Total	196 (40.8 %)	124 (25.8 %)	141 (29.4 %)	461

Regional anaesthesia was used in combination with general anaesthesia in 95 (19.9 %) cases, most commonly in orthopaedic surgery (39, 41.5 % of orthopaedic cases) and colorectal surgery (16, 35.6 % of colorectal cases). There were no significant differences IOH (>20 % of pre-induction systolic baseline) incidence in patients who received combined regional and general anaesthesia techniques in comparison with those who received general anaesthesia alone (*χ*^2^ = 0.3634). Additionally, there were no significant differences in the occurrence of intraoperative systolic hypotension between the different types of regional anaesthesia (*χ*^2^ = 0.5774).

Depth of anaesthesia monitoring was used in 46 (9.6 %) patients in 11 (44.0 %) hospitals, although 24 (52.2 %) patients’ data came from 2 hospitals. Twenty-nine (63.0 %) patients had intraoperative total intravenous anaesthesia (TIVA) and 17 (37.0 %) had inhalational anaesthesia. In those patients where DOA monitors were used, 37 (80.4 %) used BIS and 9 (19.6 %) used Entropy. Thirty-seven (80.4 %) of these patients had a DOA scores <40. In 33 (89.2 %) patients, this was combined with a systolic blood pressure reduction greater than 20 %. Twenty-seven (73.0 %) patients had a DOA score <40 for longer than 20 min.

## Discussion

We have demonstrated that IOH, as described by current definitions, is extremely common in the centres studied. Although this did vary dependant on definition, a large proportion (39 %) experienced a drop ≥40.1 %, a degree of IOH that has previously been associated with excess stroke, myocardial ischaemia and kidney injury. DOA monitoring was not used as often as suggested by NICE guidelines (National Institute for Health and Clinical Excellence 2012). However, when it was used, DOA scores less than 40 were common and this was frequently accompanied by hypotension. The formation of a trainee network to complete this project permitted the rapid completion of an audit project across a large number of hospitals within a defined geographical area.

Our results are consistent with those from ASAP in patients having proximal femoral fracture repair (ASAP collaboration team 2014), which reported IOH affected 56–89 % of patients dependant on which definition was used. IOH prevalence depends on the threshold used (Bijker et al. 2007) (relative reduction or absolute value) and component (systolic, diastolic or mean pressure), there being no universally agreed definition. We used the AAGBI definition of hypotension (Griffiths et al. 2014), a 20 % reduction in systolic blood pressure from preoperative baseline. This is a broad definition and therefore may generate high rates of IOH, but it is a suitably cautious physiological value for the older patient set by a national organisation that produces robust guidance. One might expect a large difference between our findings and those from ASAP, as the frailty of the hip fracture population may predispose to hypotensive episodes. However, we demonstrate that IOH in excess of present recommendations is a wider feature commonly affecting older surgical patients. This is also consistent with large international studies (Bijker et al. 2009) indicating it is not an issue isolated to this investigation.

The risk of harm from IOH relates to both degree and total duration, with elderly patients less able to tolerate any period of hypotension (Bijker et al. 2009). Mean arterial blood pressure less than 55 mmHg have been linked to increased morbidity in several large studies (Sun et al 2015; Walsh et al. 2013), and we found that this affected a quarter of our population. Our audit adds to these studies by demonstrating that despite national standards, both greater degrees of hypotension and duration of hypotension are common in elderly patients. Invasive arterial monitoring was used in approximately one in five patients, in keeping with AAGBI recommendations (Griffiths et al. 2014), perhaps suggesting anaesthetists recognise frailty in older patients and more readily choose invasive monitoring. However, many other practical strategies might also be employed to reduce IOH: cautious induction (Griffiths et al. 2014; Pandit et al. 2014), minimisation of anaesthesia dose guided by DOA monitoring (Willingham et al. 2015; Pandit et al. 2014, Ballard et al 2012), guided intraoperative fluid resuscitation (Pearse et al. 2014) and individualised alarm settings. Whether treatment for IOH can be effective at reducing morbidity remains to be examined in prospective studies, but the high prevalence of IOH below thresholds associated with complications (>40 % drop, MAP <55 mmHg) strongly suggests that additional treatment has the potential to improve outcome in a considerable number of patients.

Several studies have reported that combined low DOA scores (<40) and hypotension are associated with harm (Sessler et al. 2012; Willingham et al. 2014), although this remains a topic of debate and the ‘triple low’ phenomenon has been recently both questioned (Kertai et al. 2014) and supported (Willingham et al. 2015). Almost three quarters of patients whose DOA was monitored in our study had a combined ‘double low’ (concomitant BIS <40 and hypotension). Whilst we acknowledge that this was a small number of patients (33), it suggests that the technology is not being applied in an effort to minimise anaesthetic dose or lack of confidence to reduce dose below certain thresholds based on this technique. Whilst NICE guidelines on DOA monitoring have provoked some criticisms (Smith et al. 2013), particularly around who constitutes the ‘higher risk’ category, older people are well recognised to have an increased risk of perioperative complications. Subsequent to this, both the AAGBI (Griffiths et al. 2014) and Fifth National Audit Project of the Royal College of Anasthetists (NAP5) report (Pandit et al. 2014) endorsed DOA monitoring to avoid excessively deep anaesthetic states and associated hypotension, suggesting a rationale for more widespread use in the elderly.

PLAN is a trainee-led collaborative research and audit network supported by the London Academy of Anaesthesia. The structure is similar to other trainee networks (South West Anaesthetic Research Matrix 2014); projects are proposed and voted for by members and then coordinated by the proposer supported by a core committee. Our sample of 481 patients is sufficient to assess the common intraoperative targets measured and the feasibility of delivering this investigation at a larger scale, when outcomes data could also be collected. International studies have typically used retrospective analysis of electronic records; however, this is unavailable in the majority of UK departments. Trainees collected data based in recovery ensuring capture of all cases. Our aim was to assess adherence to targets rather than outcomes (which have been established by other investigators); therefore, we did not collect comprehensive patient and treatment details or postoperative outcomes, all of which potentially limit the scope of our findings. The high rate of paper anaesthetic charts in our study may cause some hypotensive episodes to be missed due to ‘smoothing’ and inaccuracies in handwritten records (Van Schalkwyk et al. 2011), meaning the true rate may actually be higher than reported. However, the written record is likely to still reflect the thresholds the anaesthetist considers important even if it deviates from electronic notes, and this is the key information of interest when informing an improvement strategy. There was variety in the quantity of data submitted from the different hospitals, and we did not assess the number or seniority of anaesthetists delivering care; therefore, our results might possibly disproportionately reflect the practice of a smaller sample of practitioners.

## Conclusions

We have demonstrated that IOH is widely prevalent in elective and unscheduled surgery in older patients in a UK setting. DOA monitoring, which might be used to minimise excessively deep anaesthesia and associated hypotension, is infrequently used. Our results suggest the need for a larger national UK investigation of practice to further clarify the clinical threshold employed for treatment of IOH in the context of patient factors and outcome. Although prospective interventional studies are required to establish an effect of tighter blood pressure control, it is unlikely that a drop of >40 % should be tolerated. Greater awareness of this issue may enable local quality improvement and improved outcomes. National studies using trainee networks have the potential to deliver these goals.

## Abbreviations

AAGBI: Association of Anaesthetists of Great Britain and Ireland
IOH: intraoperative hypotension
NAP5: Fifth National Audit Project of the Royal College of Anasthetists
NCEPOD: National Confidential Enquiry into Patient Outcome and Death
NICE: National Institute for Health and Clinical Excellence
